# Unusual Site of Oral Sarcocystosis in the Tongue

**DOI:** 10.7759/cureus.25912

**Published:** 2022-06-13

**Authors:** Thiviya Muthusamy, How Kit Thong, Primuharsa Putra Sabir Husin Athar, Aidayanti Daud

**Affiliations:** 1 Otolaryngology, Hospital Shah Alam, Shah Alam, MYS; 2 Otolaryngology - Head and Neck Surgery, Hospital Sultan Ismail, Johor Bahru, MYS; 3 Otolaryngology - Head and Neck Surgery, Kumpulan Perubatan Johor (KPJ) Healthcare University College, Nilai, MYS; 4 Otolaryngology - Head and Neck Surgery, Kumpulan Perubatan Johor (KPJ) Seremban Specialist Hospital, Nilai, MYS; 5 Otolaryngology - Head and Neck Surgery, Kumpulan Perubatan Johor (KPJ) Healthcare University College, Seremban, MYS; 6 Otolaryngology, Hospital Teluk Intan, Teluk Intan, MYS

**Keywords:** tongue, parasite, oocyst, protozoa, sarcocystis

## Abstract

*Sarcocystis *is an intracellular protozoan parasite that manifests as a sarcocyst within the muscle fibers of an intermediate host. *Sarcocystis *commonly affects animals; in fact, cases of sarcocystosis involving human hosts are rare and often undiagnosed. The two types of *Sarcocystis* species that may infect and utilize humans as a definitive host are *S. hominis* and *S. suihominis*, both of which predominantly involve the gastrointestinal system. The low prevalence of intestinal sarcocystosis among humans is rarely accompanied by symptoms, except for individuals who ingest large amounts of the parasite. This study presents an unusual case of tongue sarcocystosis, a site that has not been previously reported, that was treated successfully with oral Albendazole for two weeks.

## Introduction

*Sarcocystis *is a genus of intracellular protozoan parasites that predominantly affect mammals and reptiles. *Sarcocystis *spp. fall under the family *Sarcocystidae *and the phylum *Apicomplexa*. *S. hominis* and *S. suihominis* use humans as definitive hosts and require two hosts to complete their life cycle, which includes sexual reproduction in the intestine and asexual reproduction in the tissue [1]. The prevalence of sarcocystosis among humans varies by geographic region; it affects 1.1%-10.4% of the population in Europe, 0.4%-23.2% in Asia, and 0.5% in Australia. Furthermore, Wong et al. reported a high prevalence of skeletal muscle sarcocystosis; 21 of the 100 tongues they examined contained *Sarcocystis *[2]. *Sarcocystis *spp. infections in humans are usually asymptomatic and self-limited [1]. To date, very few studies have been written on such infections. However, the incidence of muscle sarcocystosis in humans is thought to be underreported since oocysts are often present in small numbers and difficult to detect [3]. This study reports a rare case of sarcocystosis involving the human tongue.

## Case presentation

**Figure 1:**
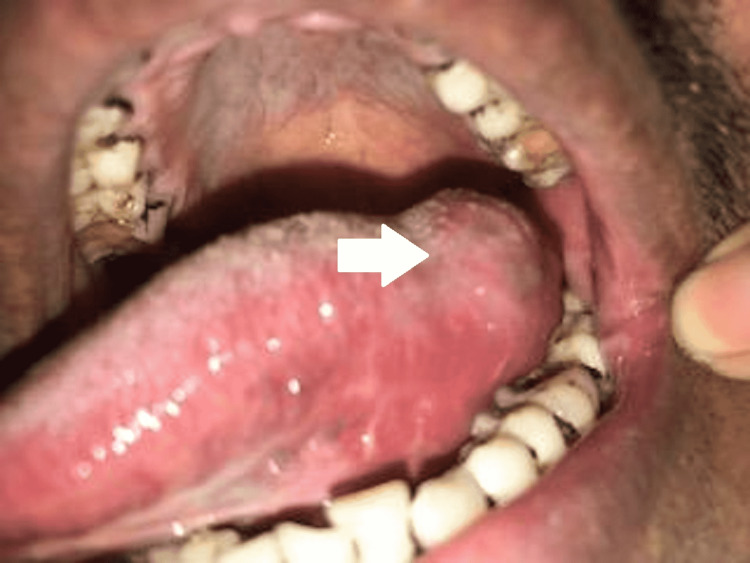
Intraoral picture showing left lateral tongue swelling (white arrow)

A 36-year-old male with an unremarkable medical history presented to our outpatient clinic; his chief complaint was progressively enlarging left lateral tongue swelling for the past four months. It was accompanied by odynophagia and pain while chewing. He denied dysphagia, dyspnea, or any constitutional symptoms. The patient had been working in the latex industry for six years with no history of tobacco smoking or alcohol consumption. Intraoral examination revealed soft swelling of the left lateral tongue measuring approximately 2 cm x 2 cm (Figure 1); additionally, the surface was irregular and tender on palpation.

**Figure 2:**
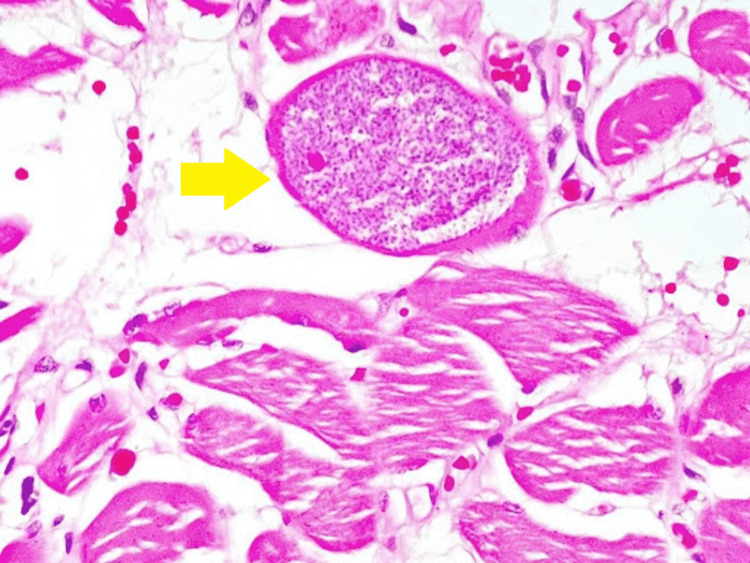
A Sarcocystis oocyst with elongated bradyzoites within the tongue muscle (yellow arrow)

Other areas of the oropharynx and oral cavity were normal, and no cervical lymph nodes were palpable. A biopsy of the lesion was performed under local anesthesia, and a histopathological examination (Figure 2) revealed the presence of intramuscular oocyst with elongated bradyzoites, which was consistent with sarcocystosis. Routine blood examination, including complete blood count, liver enzymes, renal function test, and creatine kinase, was found to be normal. He was started on oral Albendazole 400 mg once daily for two weeks, and his subsequent follow-up showed resolution of the tongue swelling.

## Discussion

Human skeletal muscle sarcocystosis is a rare condition that is diagnosed based on clusters of clinical criteria, including persistent myalgia, episodic weakness, subcutaneous nodules, and dermatomyositis. Laboratory findings of eosinophilia and elevated creatine kinase are also indicative of sarcocystosis. In certain cases, such clinical findings are linked to a history of traveling to known endemic locations. Moreover, sarcocysts in muscle biopsies can be readily identified by microscopic examination of hematoxylin- and eosin-stained sections [1]. Tests such as the enzyme-linked immunosorbent assay (ELISA) and the immunofluorescence antibody test (IFAT) can be used to detect sarcocystosis in humans [4]. Incidentally, our patient originated from Nepal and claimed that the swelling appeared after his arrival in Malaysia. However, we speculated that the patient acquired the infection in Nepal, a country where this parasitic infection is endemic. Previous studies by Rana et al. reported a high prevalence of *Sarcocystis *spp. in Nepal; consequently, most human infections occurred by ingesting raw or inadequately cooked meat of infected animals [5].

As previously mentioned, humans are one of the definitive hosts for *S. hominis* and *S. suihominis*, both of which are acquired by eating undercooked parasite-infected meat. This parasite reproduces sexually in the human intestine, which can cause symptoms of acute gastroenteritis; however, most infections remain asymptomatic [1]. Humans may also become an accidental intermediate host for one or more of the 130 known *Sarcocystis *spp. by ingesting oocysts or sporocysts in contaminated food or water [6]. In our case, since the patient denied eating raw or undercooked meat, he may have acquired the infection from contaminated drinking water. Regardless of the source, the involvement of the tongue muscle was a peculiar phenomenon.

Sarcocystosis is usually a self-limited and asymptomatic disease. To date, there are no definitive or specific treatment guidelines for human *Sarcocystis *spp. infections. Such infections can be prevented by thoroughly cooking or freezing meat to kill the bradyzoites embedded in the tissue. Despite the lack of standard treatment regimens [7], our patient responded well to oral Albendazole 400 mg per day for two weeks.

## Conclusions

In summary, sarcocystosis in humans usually affects the gastrointestinal tract and rarely involves the skeletal muscle. Our report highlights the importance of otolaryngologists remaining vigilant and scrutinizing patients with a history of traveling to a region where sarcocystosis is endemic. While this disease is usually self-limited, treatment with oral Albendazole should be considered for symptomatic cases.

## References

[1] Fayer R (2004). Sarcocystis spp. in human infections. Clin Microbiol Rev.

[2] Wong KT, Pathmanathan R (1992). High prevalence of human skeletal muscle sarcocystosis in south-east Asia. Trans R Soc Trop Med Hyg.

[3] Fayer R, Esposito DH, Dubey JP (2015). Human infections with Sarcocystis species. Clin Microbiol Rev.

[4] Thomas V, Dissanaike AS (1978). Antibodies to Sarcocystis in Malaysians. Trans R Soc Trop Med Hyg.

[5] Rana HB, Manandhar KD (2016). Studies on the prevalence of zoonotic important protozoan parasite Sarcocystis sp. in Nepal. J Adv Acad Res.

[6] Lau YL, Chang PY, Tan CT, Fong MY, Mahmud R, Wong KT (2014). Sarcocystis nesbitti infection in human skeletal muscle: possible transmission from snakes. Am J Trop Med Hyg.

[7] Nimri L (2014). Unusual case presentation of intestinal Sarcocystis hominis infection in a healthy adult. JMM Case Rep.

